# The Natural Killer–Dendritic Cell Immune Axis in Anti-Cancer Immunity and Immunotherapy

**DOI:** 10.3389/fimmu.2020.621254

**Published:** 2021-02-03

**Authors:** Erin E. Peterson, Kevin C. Barry

**Affiliations:** Translational Research Program, Public Health Sciences Division, Fred Hutchinson Cancer Research Center, Seattle, WA, United States

**Keywords:** cancer immunology, immunotherapy, natural killer cell, dendritic cell, innate immunity

## Abstract

Natural killer (NK) cells and dendritic cells (DCs) are crucial mediators of productive immune responses to infection and disease. NK cells and a subtype of DCs, the type 1 conventional DCs (cDC1s), are individually important for regulating immune responses to cancer in mice and humans. Recent work has found that NK cells and cDC1s engage in intercellular cross-talk integral to initiating and coordinating adaptive immunity to cancer. This NK cell–cDC1 axis has been linked to increased overall survival and responses to anti-PD-1 immunotherapy in metastatic melanoma patients. Here, we review recent findings on the role of NK cells and cDC1s in protective immune responses to cancer and immunotherapy, as well as current therapies targeting this NK cell–cDC1 axis. Further, we explore the concept that intercellular cross-talk between NK cells and cDC1s may be key for many of the positive prognostic associations seen with NK cells and DCs individually. It is clear that increasing our understanding of the NK cell–cDC1 innate immune cell axis will be critical for the generation of novel therapies that can modulate anti-cancer immunity and increase patient responses to common immunotherapies.

## Introduction

Natural killer (NK) cells are important innate immune effectors that belong to the family of innate lymphoid cells (ILCs). In humans, NK cells are defined by the expression of CD56 (CD3^–^CD56^+^), while the expression of NK1.1 and NKp46 (CD3^–^NK1.1^+^NKp46^+^) define NK cells in mice. Human NK cells can be divided into two subsets—highly cytotoxic CD56^dim^CD16^high^ cells located primarily in the peripheral blood, and cytokine-producing CD56^high^CD16^dim^ cells found predominately in secondary lymphoid organs [rev. in (1, 2)]. Similar functional classes of NK cells are found in mice and defined by the expression of TNF-receptor superfamily member CD27 and the integrin CD11b/Mac-1 [rev. in (1, 2)]. NK cells are heterogeneous and plastic in nature, which leads to variable expression of a number of markers (CD16, natural cytotoxicity receptors, and others) that can be used to further classify NK cells based on their differentiation, tissue location, and microenvironmental cues [rev. in (3, 4)].

NK cell effector functions consist of direct cytotoxicity to infected, transformed, and/or physiologically stressed cells [rev. in (5, 6)] and modulation of immune responses through the production of cytokines and chemokines [rev. in (2, 7)]. These functions are regulated by an intricate balance of germline-encoded inhibitory and activating receptors [rev. in (2, 4)]. Inhibitory receptors expressed by NK cells include Ly49-type inhibitory receptors (in mice), killer immunoglobulin-like receptors (KIRs; in humans), and the CD94-NKG2A heterodimer (in humans and mice) [rev. in (2)]. Activating receptors expressed by NK cells include the natural cytotoxicity receptors (NCRs) NKp46, NKp44, and NKp30, the lectin-like type 2 transmembrane receptor NKG2D, DNAX accessory molecule 1 (DNAM1/CD226), and adhesion molecule lymphocyte function-associated antigen-1 (LFA-1) [rev. in (2)]. These receptors balance inhibitory signals delivered through the recognition of self MHC-I molecules and activating ligands that are upregulated in response to cellular stress, infection, or transformation [rev. in (2, 8, 9)]. Additionally, NK cell function may be regulated by the immune checkpoint molecules PD-1, CTLA-4, TIGIT, LAG3, and TIM-3 [rev. in (4, 10)].

NK cells are integral to antiviral responses, anti-cancer immunity [rev. in (2, 7, 11, 12)], and have a role in cancer prevention (13). NK cell abundance in the tumor microenvironment (TME) is associated with greater overall survival in patients with melanoma (14–18), hepatocellular carcinoma (19), pulmonary adenocarcinoma (20), renal cell carcinoma (21), gastric cancer (22), breast cancer (23), squamous cell lung cancer (24), non-small cell lung cancer (25), and neuroblastoma (26). The protective immunity provided by NK cells is controlled by their direct cytotoxicity and the production of immunomodulatory cytokines and chemokines that sculpt local and distant immune cell responses [rev. in (2, 4, 7, 11, 12, 27)]. This critical association of NK cells with controlled tumor growth and metastasis highlights their role as dynamic anti-cancer agents [rev. in (4, 7)].

Dendritic cells (DCs) are an innate immune myeloid cell that provide an essential link between the innate and adaptive immune responses. As specialized antigen-presenting cells DCs play a key role in initiating T cell-mediated antigen-specific immunity and tolerance [rev. in (28)]. DCs perform this role by continuously sampling and presenting antigens to T cells *via* major histocompatibility complex (MHC) I and II, a process that is greatly increased upon activation [rev. in (29, 30)]. DC functions are shaped by the integration of environmental cues sensed by pattern recognition (PRRs) and cytokine receptors [rev. in (29, 31)].

Conventional DCs can be classified most simply as cDC1 or cDC2, both of which express CD11c and MHC-II in humans and mice [rev. in (32)]. cDC1s and cDC2s are defined by, and require, distinct transcription factors and cell surface markers, possess differential growth factor requirements, and, critically, undertake distinct functions (29). cDC1s rely on the transcription and growth factors BATF3, IRF8, BCL6, ID2, and FLT3L for development, and can be defined by expression of the chemokine receptor XCR1 and the C-type lectin endocytic receptor CLEC9A [rev. in (29, 32–34)]. Human cDC1s can be further identified by BDCA3 expression, while murine cDC1s can be defined as CD103^+^ or CD8α^+^ populations [rev in (29)]. cDC2s, on the other hand, depend on IRF4 and ZEB2 for development and express CD11b and CD172a [rev. in (29, 32)]. Classically, cDC1s are thought to induce robust CD8^+^ T cell responses, while cDC2s are thought to be more important for CD4^+^ T cell responses (29). cCD1s and cDC2s both play roles in anti-cancer immunity [rev. in (32) (35)], but we will focus on the protective effects of cDC1s in this review.

**Figure 1:**
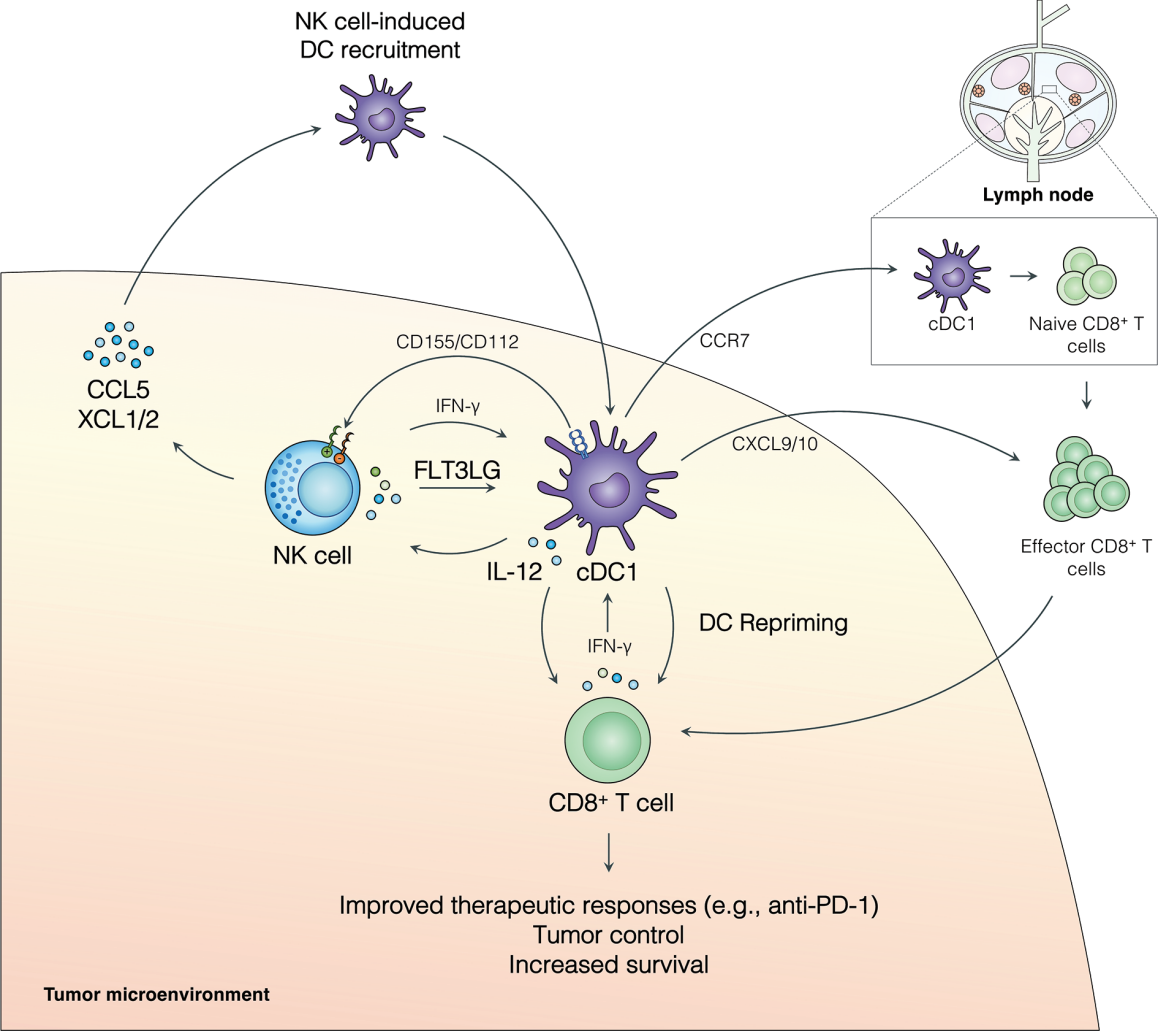
The NK cell-cDC1 axis modulates the TME to boost immune responses to cancer. NK cell production of the chemokines CCL5, XCL1, and XCL2 recruits cDC1s to the tumor. Additionally, FLT3L produced by NK cells increases survival and/or differentiation of cCD1s in the tumor. IFN-γ produced by NK cells enhances cDC1 antigen presentation and maturation and leads to cDC1 production of IL-12, which can increase NK cell activity. cDC1s can further modulate NK cell activity through the expression of CD155 and/or CD112 which can signal through inhibitory receptors (TIGIT and CD96) or activating receptors (CD226/DNAM1) expressed on NK cells. Activated cDC1s in the TME upregulate CCR7 and migrate to the tumor-draining lymph node, where they activate naive CD8^+^ T cells. Effector CD8^+^ T cells are recruited to the TME, at least in part, by cDC1-produced CXCL9/10 and, critically, undergo local restimulation by cDC1s. Repriming of CD8^+^ T cells in the TME increases tumor control, patient survival, and improves responses to anti-PD-1 immunotherapy.

The presence of cDC1s in the TME is correlated with improved clinical outcomes in numerous cancers and serves as a strong biomarker for responsiveness to anti-PD-1 immunotherapy in metastatic melanoma patients (16, 17, 26, 32, 36–38). While cDC1s are rare in human and murine tumors, they efficiently cross-present exogenous antigens to CD8^+^ T cells, are capable of initiating *de novo* cytotoxic CD8^+^ T cell responses after migrating to the tumor-draining lymph node, and play an integral role in re-priming CD8^+^ T cells directly in the TME [rev. in (29, 32, 36)] (**Figure 1**). Further, cDC1s can contribute to T helper (Th) 1 cell polarization of naive CD4^+^ T cells [rev. in (29, 32, 36)]. Augmenting cDC1 frequency in the tumor has resulted in enhanced tumor responses (16, 17, 32, 39). Alternatively, the *in vivo* depletion of cDC1s is associated with failed tumor rejection, immune escape, and an inability to respond to multiple T-cell immunotherapies—such as immune checkpoint blockade and adoptive T cell therapy [rev. in (29, 32)]. Accordingly, cDC1s provide essential roles in anti-cancer immune responses and offer promising immunotherapeutic targets against cancer.

## The Natural Killer–Dendritic Cell Innate Immune Axis in Cancer

There is a rich literature surrounding the individual functions of NK cells and DCs in anti-tumor immunity (2, 7, 16, 17). Recent work supports an integral role for NK cells in shaping DC maturation and promoting DC recruitment, retention, and/or survival in the tumor (2, 7, 16, 17). It is well known that NK cells can perform DC editing, a quality control process in which activated NK cells selectively kill immature DCs to ensure successful T cell priming by mature, immunogenic DCs [rev. in (2, 4)]. NK cell editing of DCs established a direct, functional relationship between NK cells and DCs [rev. in (2, 4)].

Emerging evidence indicates that NK cell-cDC1 interactions have a profound effect on anti-cancer immunity (**Figure 1**). NK cells promote the recruitment of cDC1s into the TME through the production of the chemokines CCL5, XCL1, and XCL2 (16). This pathway is abrogated by the presence of tumor-derived prostaglandin E2 (PGE_2_), which leads to impaired NK cell function and downregulation of CCR5 and XCR1 receptors on cDC1s (16). *CCL5* expression has further been linked to NK cells and the abundance of cDC1s in neuroblastoma patient samples (26). NK cells also produce FLT3LG, the formative cytokine for cDC1s, in the TME (17) (**Figure 1**). *FLT3LG* expression and NK cell abundance in the tumor are correlated with increased cDC1 levels, better overall survival, and increased responses to anti-PD-1 immunotherapy in metastatic melanoma patients (17). Further, cDC1s and NK cells were positively correlated with *FLT3LG* expression, T cell infiltration, increased survival, and the expression of checkpoint molecules (PD-1 and PD-L1) in the tumors of glioblastoma patients (26). NK cell production of FLT3LG may increase cDC1 survival in the TME, but other mechanisms, such as increasing the differentiation of precursor DCs (pre-DCs), remain possible (17). The importance of FLT3L control of cDC1s in the tumor was further demonstrated in a recent murine model of pancreatic ductal adenocarcinoma (PDAC) where FLT3L and anti-CD40 combination therapy restored cDC1 infiltration, improved CD8^+^ T cell and Th1 control of tumor growth, and boosted responses to external radiation therapy (40, 41). These data suggest that NK cells play an important role in recruiting and retaining cDC1s in the tumor, which subsequently activate protective anti-tumor CD8^+^ T cell responses (**Figure 1**).

The NK cell-cDC1 innate immune axis is undoubtably a bidirectional relationship with cDC1s playing an important role in regulating NK cells in the tumor. The role of DCs in shaping NK cell responses in cancer has been thoroughly reviewed recently [rev. in (42)]; thus, we will provide a brief description of these mechanisms and how they may affect the NK cell-cDC1 axis. Activated DCs produce IL-12, which stimulates NK cells and anti-tumor T cell immunity [rev. in (32)] (43) (**Figure 1**). Further, IL-12 production by tumor-infiltrating DCs is required for effective anti-PD-1 immunotherapy responses (43). In addition to cytokines, DCs express a number of cell adhesion molecules with immune regulatory functions that can regulate NK cells [rev. in (44–47)] (**Figure 1**). CD155 and CD112 are two such molecules that are upregulated upon DC maturation and activation and can signal through the receptors CD226/DNAM1, TIGIT (T cell immunoreceptor with immunoglobulin and ITIM domains), and CD96 expressed on NK cells [rev. in (44–47)]. CD226 ligation by CD155 or CD112 can induce NK cell activation, while ligation of TIGIT inhibits NK cells (rev. in (44–47)]. Ligation of CD96 acts as an inhibitor of NK cell responses in mice, but the role of CD96 in human NK cells remains less clear (45). A recent study found that a subset of CD112-expressing DCs in the TME of human hepatocellular carcinoma interact with NK cells through CD226 and TIGIT (48). These data suggest that expression of CD155 and CD112 by DCs can modulate NK cell responses in the tumor. Importantly, NK cell expression of CD226 has been linked to the NK cell-dependent killing of immature and mature DCs expressing CD155 and CD112 (49), suggesting this pathway may play multiple roles in shaping the NK cell-cDC1 axis. The activation of NK cells by DCs (e.g., IL-12, CD226/CD155) may act as a feed-forward loop to increase DC activation through the induction of NK cell production of chemokines and cytokines, subsequently improving anti-tumor immune responses [rev. in (32)] (43). More research is needed to determine how DC-dependent stimulation or inhibition of NK cells may shape production of XCL1, XCL2, CCL5, and FLT3LG in the TME. However, a bidirectional relationship between NK cells and cDC1s in the TME clearly exists and targeting cDC1 factors that influence NK cell activity may be another tool to increase immune responses to cancer.

NK cells and cDC1s make stable and close interactions in the TME of mouse ectopic B78 melanoma tumors (17) and multiplex immunofluorescence imaging of human glioblastoma found a similar close interaction (26). Given these findings, it is intriguing to hypothesize that local concentrations of chemokines and cytokines (e.g., FLT3LG, IFN-γ, or IL-12), or receptor-ligand interactions (e.g., CD226/CD155) that require cell-cell contacts, may control the NK cell-cDC1 axis. The spatial organization of NK cells and cDC1s in the tumor may be an important factor in controlling the NK cell-cDC1 axis (17, 26), but clearly more data is needed to fully define this spatial regulation.

The NK cell-cDC1 axis is integral for controlling immune responses to cancer and is linked to increased patient survival and/or responses to immunotherapies in metastatic melanoma, head and neck squamous cell carcinoma, triple negative breast cancer, and neuroblastoma (16, 17, 26). It is important to note that in certain tumor settings NK cells have been shown to have inhibitory effects on DC functions (50–52) and DCs can have inhibitory effects on NK cell functions [rev. in (44–47)]. These findings suggest that there may be tumor-specific regulation of the NK cell-cDC1 axis and highlight the need for more detailed studies of this innate immune axis across other cancer indications. The data presented here suggest that a better understanding of the mechanisms that influence the bidirectional relationship of NK cells and cDC1s in the tumor could be used to enhance existing therapies or reveal new therapeutic avenues to protect patients from cancer.

## NK Cell Regulation of cDC1s in Pre-Clinical Models of Cancer

The NK cell-cDC1 axis has been defined within the TME and, as such, these cells are susceptible to tumor-induced immune suppression [rev. in (53–55)]. Conditions in the TME can lead to impairment of antigen presentation, activation of negative costimulatory signals (i.e., immunological checkpoints), and production of immunosuppressive and pro-apoptotic factors [rev. in (53–55)]. Metabolic restrictions within the tumor are also known to inhibit immune responses [rev. in (56)]. Namely, nutrient and oxygen deficiency and increased concentration of metabolic products (e.g., adenosine, lactic acid, retinoic acid) (57–59) can pose significant challenges to infiltrating immune cells [rev. in (32)]. As such, diverse strategies have been explored to improve effector cell responses in cancer. Importantly, the NK cell-cDC1 axis must be studied in the context of these suppressive signals in the TME.

A number of studies have shown that shifting the cytokine milieu or metabolic factors in the TME to modulate NK cells activity can lead to protective immune responses to cancer [rev. in (2, 7)]. Here, we will review recent studies exploring these novel mechanisms and, further pose the question: are the protective responses seen by targeting NK cells, at least partially, due to changes in NK cell regulation of cDC1s in the tumor?

### Adenosine 2A Receptor

Adenosine is an immunosuppressive metabolite present at high levels in the TME [rev. in (60–63)]. Adenosine signaling through A2A adenosine receptor (A2AR) on immune cells can dampen anti-tumor immune responses [rev. in (64)]. In a recent study, A2AR signaling was shown to inhibit NK cell maturation in mice at homeostasis and in the tumor (65). Transcriptional profiling of *A2AR*-deficient NK cells revealed decreased expression of the receptor tyrosine kinase *KIT* (*CD117*) and the interleukin-18 (IL-18) receptor *IL18R1* (65). This transcriptional profile is interesting because it is opposite to a population of pro-tumorigenic KIT^+^ NK cells found to deplete peripheral pools of DCs (51). These findings suggest that inhibition of A2AR signaling in NK cells may lead to improved anti-tumor activity through a maintenance of DC populations. Further, *A2AR*-deficient NK cells were found to have enhanced maturation, maintain a proliferative advantage over wildtype NK cells, and protect against tumor development in a transplantable BRAF-melanoma tumor model (65). Taken together, these findings demonstrate that pairing A2AR antagonism with NK cell-based immunotherapies may provide a combinatorial strategy to improve therapeutic efficacy (65). Furthermore, these studies suggest that A2AR inhibition may help maintain the NK cell-cDC1 axis. Additional work will provide more insight into the role of the NK cell-cDC1 axis in the anti-tumor protection provided by A2AR antagonism.

### Interleukin-18 (IL-18)

Treatment with high doses of IL-18 induce increased inflammation (66) and improved responses to immune checkpoint blockade (67) and CAR-T cells (68). A recent study generated a decoy-resistant IL-18 cytokine (DR-18) that has high binding affinity for the IL-18 receptor, IL-18Rα, but is unable to bind to the decoy receptor, IL-18BP (69). DR-18 treatment protects animals from ectopic models of melanoma (YUMMER1.7) and colorectal cancer (MC38) (69). Further, in β2m-deficient tumors, control of tumor growth by DR-18 requires NK cells, and DR-18 treatment increases the abundance of a cluster of NK cells that produce various effector molecule transcripts (*IFNG*, *PRF1*, *GZMB*), as well as the chemokines *CCL5* and *CCL4*, in the TME (69). CCL5 production by NK cells has previously been linked to increased recruitment of cDC1s to the TME (16). These data suggest that, in certain situations, NK cells upregulate chemokines that could increase cDC1 levels in the TME in response to DR-18. However, in wildtype tumors, DR-18 acts directly on T cells in the TME and functions independently of cDC1s (69). Thus, it is intriguing to speculate that, depending on the setting, DR-18 treatment may protect against cancer in multiple ways, including by modulating the NK cell-cDC1 innate immune axis. However, it appears that direct stimulation of T cells in the TME may be the primary driver of protection, at least in the tumor models tested thus far.

### TIGIT and CD96

TIGIT and CD96 are inhibitory receptors that signal through binding the shared ligand CD155 and CD112 or CD111, respectively (45). The role of TIGIT and CD96 in immune responses to cancer has been thoroughly reviewed elsewhere (45). As such, we will focus our discussion to recent findings regarding the role of TIGIT in regulating NK cell responses to cancer.

TIGIT^+^ NK cells are found in human hepatocellular carcinoma (70), human colorectal cancer, and a number of murine tumor models, including breast cancer (4T1), melanoma (B16), colon cancer (CT26), and MCA-induced fibrosarcoma (71). Interestingly, genetic or antibody blockade of TIGIT signaling on NK cells was found to increase NK cell function and boost cytotoxic CD8^+^ T cell responses and protective T cell-mediated memory responses (71). NK cells are required for the protective effects of anti-TIGIT alone or in combination with anti-PD-L1 immunotherapy and, in fact, are partially required for the protective immune responses caused by anti-PD-L1 treatment alone (71). Protective responses induced by anti-TIGIT treatment partially require IFN-γ, and a role for direct NK cell cytotoxicity cannot be ruled out (71). It remains to be seen if these protective effects of NK cells in response to anti-TIGIT therapy function through the modulation of cDC1s in the TME, but, given the strong effects this NK cell-dependent treatment has on CD8^+^ T cells, it is an intriguing hypothesis.

## Modulation of the NK Cell-cDC1 Axis in the Clinic

NK cell-directed immunotherapies show great promise in the clinic [rev. in (2, 7)]. However, it is unknown if current NK cell-based therapies function through increasing cDC1s in the tumor. Here, we will discuss a recent clinical treatment, the intratumoral electroporation of a plasmid encoding IL-12 (tavokinogene telseplasmid; “tavo”) (72–74), and explore its potential role in shaping the NK cell-cDC1 axis.

Interleukin-12 (IL-12), regulates NK cell and T cell responses, promotes Th1 polarization, and is a potent regulator of immune responses to infection and cancer [rev. in (75, 76)]. Systemic treatment with recombinant IL-12 (rIL-12) has shown efficacy in animal models of cancer, but these treatments are associated with modest clinical response and serious adverse events in patients [rev. in (77)]. Alternatively, intratumoral electroporation of tavo (IL-12) was found to be safe in a Phase I clinical trial, demonstrated preliminary efficacy by increasing intratumoral IL-12 and IFN-γ, and led to remission in several patients (74). Two recent Phase II clinical trials of tavo (IL-12) electroporation found that this treatment leads to an increase in NK cell and cDC1-related transcripts in the tumor, an increase in CD8^+^ T cells in the tumor, and activation of systemic immune responses in treated patients (72, 73). In these studies, it was proposed that intratumoral electroporation of tavo (IL-12) appears to boost NK cell abundance in the tumor, which leads to an increase in abundance of protective cDC1s, increased T cell responses, and, in some patients, durable responses to treatment (72). It was further shown that intratumoral electroporation of tavo (IL-12) can increase immune infiltration in poorly infiltrated metastatic melanoma tumors and subsequently increase patient responses to anti-PD-1 immunotherapy (73).

These studies suggest that targeting the NK cell-cDC1 innate immune axis with the electroporation of IL-12 into the tumor may have efficacy as a single agent and may shape the TME to be more responsive to anti-PD-1 immunotherapy. It is important to note that electroporation of tavo (IL-12) could act directly on T cells to shape immune responses to melanoma, and thus more basic and clinical research is needed to fully elucidate the mechanisms by which this treatment is providing protection to patients. However, these correlative findings are consistent with IL-12 increasing NK cell activity in the TME and subsequently boosting cDC1 abundance and CD8^+^ T cell responses to the tumor.

## Conclusion

As highlighted in this review, NK cells and cDC1s have a rich literature demonstrating their important individual roles in supporting protective immune responses to cancer. We propose that at least some of these protective roles are related to the cross-talk between NK cells and cDC1s. We have provided evidence that the NK cell-cDC1 axis is a bidirectional relationship with each cell type shaping the responses of the other. We also highlight pre-clinical and clinical studies that suggest that targeting the NK cell-cDC1 axis may provide novel pathways to increase immune responses to cancer. We propose that the NK cell-cDC1 axis should be considered in future studies exploring the individual association of these cell types in controlling immune responses to cancer. Clearly, the NK cell-cDC1 innate immune axis has important roles in shaping immune responses to cancer and future studies are needed to determine exactly how this axis can be targeted and manipulated as a tool to boost immune responses to cancer.

## Author Contributions

EP and KB conceived, wrote, and edited the manuscript. All authors contributed to the article and approved the submitted version.

## Conflict of Interest

The authors declare that the research was conducted in the absence of any commercial or financial relationships that could be construed as a potential conflict of interest.
